# Patient Engagement and Patient Experience Data in Regulatory Review and Health Technology Assessment: Where Are We Today?

**DOI:** 10.1007/s43441-025-00770-6

**Published:** 2025-04-10

**Authors:** Neil Bertelsen, Elisabeth Oehrlein, Bronwyn Lewis, Tiffany Westrich-Robertson, Jim Elliott, Tom Willgoss, Nidhi Swarup, Ify Sargeant, Oishika Panda, Maria M. Marano, Hayley Chapman, Nicholas Brooke

**Affiliations:** 1 HTAi Patient & Citizen Involvement in HTA Interest Group and Neil Bertelsen Consulting, Berlin, Germany; 2 Applied Patient Experience, Washington, DC USA; 3 Patient Engagement, Boehringer Ingelheim, Frankfurt, Germany; 4 AiArthritis (International Foundation for Autoimmune & Autoinflammatory Arthritis), St Louis, MO USA; 5 Independent Expert, Dinas Cross, Wales, UK; 6 Roche Products Ltd, Welwyn Garden City, UK; 7 Alliance of Patients’ Organizations Singapore Ltd and Consumer Engagement & Education Panel, Agency for Care Effectiveness, Ministry of Health, Singapore, Republic of Singapore; 8 Twist Medical, Burlingame, CA USA; 9 Patient Focused Medicines Development, Brussels, Belgium

**Keywords:** Patient experience data, Patient engagement, Health technology assessment, Regulatory assessment, Real-world evidence

## Abstract

**Background:**

As healthcare stakeholders aim to support patient-centered care, patients play an increasingly important role in pharmaceutical and medical technology development and healthcare decision-making. Patient engagement (PE), patient experience data (PED), and meaningful integration of PE to enrich PED have been evolving rapidly. This landscape review focuses on emerging PE/PED practices and guidelines in 2023.

**Methods:**

References published between January–December 2023 on the use of PE and PED from health technology assessment (HTA) and regulatory bodies in different countries, three peer-reviewed journals, and referred resources from collaborators were analyzed. These references were compared with those in our previous publication (August 2021–January 2023, 17-month period).

**Results:**

Overall, 28 references from HTA/regulatory bodies, 26 from peer-reviewed articles, and 17 referred resources were identified. Eight references on PE and PED integration (PE + PED) were identified in 2023 from HTA/regulatory bodies, compared with none in the previous 17-month analysis. Emerging trends on the role of PE, PED, and real-world evidence in HTA/regulatory deliberations, transparency and geographic variations in the use of such evidence and practices, and gaps thereof have been highlighted.

**Conclusions:**

The increase in PE, PED, and PE + PED references worldwide in 2023 versus the prior 17-month analysis suggests accelerated adoption of PE + PED practices. However, a need remains for comprehensive, actionable guidance on best practices for use of PE and PED for harmonization and incorporation into HTA/regulatory processes. Patient input—essential for evidence-based decision-making—provides valuable insights that enhance care quality, treatment relevance and effectiveness, and builds trust and sustainability.

**Supplementary Information:**

The online version contains supplementary material available at 10.1007/s43441-025-00770-6.

## Introduction

Healthcare systems are profoundly shaped by the recommendations of regulatory and health technology assessment (HTA) bodies about which pharmaceuticals and medical technologies are made available to patients. Regulatory bodies focus on evidence to assess the quality, safety, and efficacy of a health intervention; HTA bodies compare the effectiveness of new interventions with current approaches already used within national healthcare systems to evaluate costs and benefits [1, 2]. Such evidence is predominantly generated by the developers of pharmaceuticals and medical technologies (“evidence generators”) [2, 3].

Evidence-based healthcare systems, with the goal of supporting patient-centered care, deliver better health outcomes when decisions are meaningfully informed by patient perspectives, needs, and priorities [4]. As experts in living with a condition, and as the ultimate users of health interventions, patients and their caregivers are uniquely positioned to articulate their experiences, preferences, and unmet needs [5–7]. There is increasing global recognition of the value of patient engagement (PE) and patient experience data (PED) in the pharmaceutical and, to some extent, medical technology sectors, as well as in regulatory/HTA processes, to improve the quality of recommendations and patient access to new treatments of value to them [2, 8, 9].

There is an array of definitions for PE and PED in the literature. However, in this article, PE is defined as bidirectional, meaningful interactions that involve patients/caregivers in healthcare decision-making processes across the product’s lifecycle, to ensure that such decisions reflect their priorities [10]. The US 21st Century Cures Act defines PED as information about patients’ lived experiences with a given disease/condition—including the physical/psychosocial impact of the disease/condition or related therapy—and their treatment preferences [11]. Such qualitative and quantitative PED can be collected via the patient community and other stakeholders, including disease research foundations, researchers, and drug/medical technology manufacturers [3, 12]. In some countries, patient organizations (POs) can input directly to the HTA review process, independent of the evidence submitted by pharmaceutical and medical technology developers [13]. PE can support PED to ensure patient-centricity of measurement, contextualization of data, increased understanding of the evidence generated, and the co-creation of the design, generation, collection and analysis of PED [14]. We refer to this approach as ‘integration of PE and PED’ (PE + PED). Meaningful PE + PED has evolved rapidly as healthcare stakeholders increasingly recognize the value of better design, generation, and analysis of this crucial information [15].

Our previous landscape review provided a snapshot of recent developments in the use of PE and PED in regulatory/HTA processes [10]. We also highlighted a pressing need for more comprehensive, universally applicable guidance on how to engage patients more fully in healthcare evidence generation and regulatory assessments. At the 77th World Health Assembly (June 2024), member states of the World Health Organization (WHO) endorsed a resolution on the sustained implementation of regular and meaningful social participation in healthcare decision-making processes. The WHO defines social participation as the empowerment of “people, communities, and civil society through inclusive participation in decision-making processes that affect health across the policy cycle and at all levels of the system” [16].

Responding to this call to action and acknowledging the rapidly accelerating evolution of practices and guidance in PE/PED, here we present a revised landscape review to inform a diverse range of stakeholders about their integration into regulatory/HTA considerations. We investigate how regulatory/HTA bodies globally are guiding best practices for generating PED and providing clarity on its use in their assessments. We also aim to identify emerging trends in the peer-reviewed literature and other miscellaneous references that may indicate increasing integration of PE and PED in HTA and regulatory deliberations.

## Methods

The previous landscape analysis [10] focused on references published August 2021–January 2023; here, references published January–December 2023 were examined. All authors of this article are active in the space for PE and PED within regulatory and HTA assessments. With all author agreement, this study was conducted in a streamlined manner and within the timeframe and budget constraints using a three-pass approach described below. The search was restricted to English language references.

### References from HTAs/Regulatory Bodies

Authors identified key HTA and regulatory institutions that are active and leading the way in thinking about PE and PED approaches. Organization websites for Canada’s Drug Agency (CDA; formerly the Canadian Agency for Drugs and Technologies in Health [CADTH]), Institute for Clinical and Economic Review (ICER), European Medicines Agency (EMA), US Food and Drug Administration (FDA), and the draft Regulation (EU) 2021/2282 on Health Technology Assessment (HTAR) were queried for the terms “patient involvement,” “patient engagement,” “patient evidence” (to include patient evidence and patient-based evidence), “patient experience data,” “patient preference study,” “patient reported” (to include patient-reported outcomes and patient-reported experience measures) to search for documents published during 2023. Organizational websites were not equipped for multi-pronged search, and each search term was applied individually. Filters such as “guidance” and “new” were used where available. Because on many sites the search terms often yielded unrelated content that could not be filtered, the total number of search hits was not easily ascertained.

Additionally, the Patient-Centered Evidence in Action (APx-PACE) database [17] was used to investigate PED, either submitted by sponsors or independently identified by FDA staff, among all new drug applications (NDAs) and biologic license applications (BLAs) approved in 2023 by the Center for Drug Evaluation and Research (CDER). These websites for HTA/regulatory bodies were included because they had published new English-language information in 2023.

### Peer-Reviewed Journals

Authors focused on three peer-reviewed journals that commonly publish on these subjects. The journals *Value in Health* (official journal of the Professional Society for Health Economics and Outcomes Research [ISPOR]) and *International Journal of Technology Assessment in Health Care* (official journal of the Health Technology Assessment International [HTAi]) were included because they focus on health economics and publish relevant research articles on HTA bodies. *Therapeutic Innovation & Regulatory Science* (official journal of the Drug Information Association [DIA]) was selected as a reliable resource on developments in the regulatory space. These three journals were queried for (“patient involvement” OR “patient engagement” OR “patient evidence” OR “patient based evidence” OR “patient preference study” OR “patient reported outcomes” OR “patient reported experience measures” OR “patient experience data” OR “qualitative evidence”) AND (“regulator” OR “HTA”) AND (“resource” OR “toolkit” OR “guidance” OR “framework”). Articles not published in calendar year 2023, and those without open access, were excluded.

### Referred Resources

Patient Focused Medicines Development (PFMD), Brussels, Belgium, is a global non-profit multi-stakeholder collaboration committed to integrating the patient voice in drug/medical technology development [18]. As such, this group is well-positioned to identify strategic areas for PE and PED at every step of healthcare technology development, including regulatory and HTA deliberations. To identify important activities for the landscape that were not captured by the aforementioned searches, references published in 2023 that discussed PE, PED, or PE + PED were collected via referrals from colleagues and collaborators and manually analyzed by the team at PFMD (Table S1) to assess for a focus on PE, PED, or PE + PED. Information sources that were likely not verified or validated by peer review (e.g., podcasts) were categorized as “gray literature” and excluded.

### Reference Categories

References included in the analysis were categorized as:

- **PE**: Discussed/included relevant information on PE, e.g. how a regulator created a patient advisory board;
- **PED**: Discussed/included relevant information on PED, e.g. journal article analyzing whether evidence generated through social media was robust enough to support insights about patient preferences;
- **PE + PED**: Discussed/included relevant information on integration of PE and PED, e.g. engaging patients to determine which PED outcome measures should be prioritized in an assessment.

## Results

**Fig. 1:**
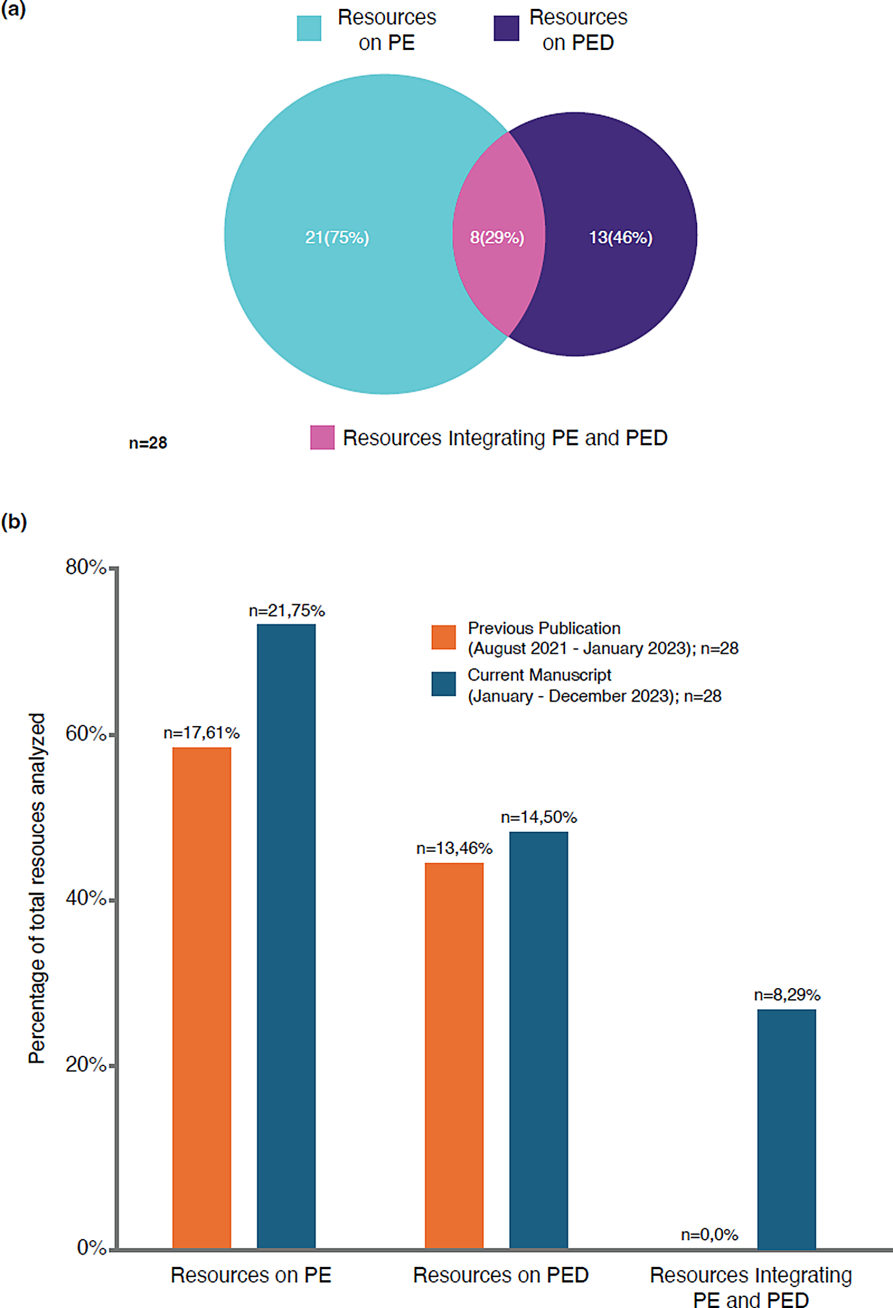
**(A)** References containing information on PE, PED, and PE + PED published by HTA and regulatory bodies in 2023 (categorical assignments were not mutually exclusive); and **(B)** comparison with numbers of references from HTA and regulatory bodies in the previous publication [10] HTA, health technology assessment; PE, patient engagement; PED, patient experience data

Altogether, 27 references published in 2023 were identified from HTAs and 18 from regulatory bodies worldwide. Of these, 18 and 10 references from HTAs and regulatory bodies, respectively, fulfilled inclusion criteria [3, 12, 19–44] (Table 1). Of the combined 28 references from HTAs/regulatory bodies, 21 (75%) discussed PE, and 13 (46%) discussed PED; many of the references considered both PE and PED and were counted more than once. Fewer references on PED were obtained from HTA bodies than regulatory organizations (8 vs. 6, respectively; Fig. S1). Unlike our previous landscape analysis [10], which also identified 28 references but found none exploring an integrated approach, for 2023 there were 8 (29%) on PE + PED in HTA/regulatory assessments (Fig. 1).

A further 26 peer-reviewed journal articles [2, 10, 45–68] (Table 2) and 16 referred resources [69–84] (Table 3) were also included.

**Table 1:** Global patient engagement (PE) and patient experience data (PED) references in regulatory and health technology assessment processes included in the analysis

Region/ country	Resource/initiative	Organization/agency	Type of organization	References on PE	References integrating PE + PED	References on PED
Australia	HTA Policy and Methods Review: Economic evaluation [19]	CHERE	HTA/University economics dept	✓		✓
Australia	HTA Policy and Methods Review: Clinical evaluation methods in HTA [20]	NHMRC Medicines Intelligence Centre of Research Excellence (commissioned by the Australian Government—Department of Health and Aged Care)	HTA/University economics dept	✓		
Australia	HTA Policy and Methods Review: Horizon scanning and early assessment [21]	NHMRC Medicines Intelligence Centre of Research Excellence (commissioned by the Australian Government—Department of Health and Aged Care)	HTA/University economics dept	✓		
Australia	HTA Policy and Methods Review: International health technology market approval, funding and assessment pathways [22]	NHMRC Medicines Intelligence Centre of Research Excellence (commissioned by the Australian Government—Department of Health and Aged Care)	HTA/University economics dept	✓		
Australia	Optimising real-world data and real-world evidence to support HTA [12]	NHMRC MI-CRE	HTA/University economics dept			✓
Canada	Pilot Expert Committee to support decision-making across the drug life cycle [23]	CDA	HTA	✓		
Canada	CADTH 2023 Symposium [24]	CDA	HTA	✓		
Canada	Revised timing for patient/clinician input [25]	CDA	HTA	✓		
Canada	Lecture: Reimagining HTA assessment [26]	CDA	HTA		✓	✓
Canada	Guidance for reporting RWE [27]	Health Canada	Regulator			✓
Europe	Practice guidance: Outcomes (endpoints) [28]	EUnetHTA	HTA	✓	✓	✓
Europe	EMA’s Regulatory Science Strategy: achievements to end of 2022 [29]	EMA	Regulator	✓	✓	✓
Europe	Guidance for EMA/EUnetHTA 21 Joint Scientific Consultation [30]	EUnetHTA	HTA	✓		
Europe	HTA stakeholder network [31]	EU HTA	HTA	✓		
Europe	Agenda for joint meeting CTTI/FDA, PEC, PCWP [32]	EMA	Regulator	✓		
Europe	Final programming 2023–2025 [33]	EMA	Regulator	✓		
Singapore	Opportunities for patient involvement [34]	ACE	HTA	✓		
US	Updated Value Assessment Framework [35]	ICER	HTA	✓	✓	✓
US	Processes for conducting value assessments [36]	ICER	HTA	✓	✓	✓
US	PFDD: Incorporating clinical outcome assessments into endpoints for regulatory decision-making [37]	FDA	Regulator	✓	✓	✓
US	Advancing HTA assessments that support health equity [38]	ICER	HTA	✓	✓	
US	Snapshot: Sickle cell disease [39]	ICER	HTA	✓		
US	Establishment of a Patient Council [40]	ICER	HTA	✓		
US	2-day webcast to enhance clinical study diversity [41]	FDA	Regulator	✓		
US	8th Annual Clinical Outcome Assessment in Clinical Trials Workshop (virtual meeting) [42]	FDA	Regulator		✓	✓
US	Press release on seeking increased funding for public health [43]	FDA	Regulator			✓
US	Press release on PFDD [44]	FDA / EIN Newswires	Regulator			✓
US	Guidance on use of RWE to support regulatory decision-making for medical devices [3]	FDA	Regulator			✓

ACE, Agency for Care Effectiveness; CADTH, Canadian Agency for Drugs & Technologies in Health; CDA, Canada’s Drug Agency; CHERE, Centre for Health Economics Research and Evaluation; CTTI, Clinical Trials Transformative Initiative; EMA, European Medicines Agency; EUnetHTA, European Network for Health Technology Assessment; FDA, Food & Drug Administration; HTA, health technology assessment; ICER, Institute for Clinical and Economic Review; MI-CRE, Medicines Intelligence Centre of Research Excellence; NHMRC, National Health and Medical Research Council; PE, patient engagement; PED, patient experience data; PE + PED, integrated PE and PED; PFDD, patient-focused drug development; PCWP, Patients’ and Consumers’ Working Party; PEC, Patient Engagement Collaborative; RWE, real-world evidence

**Table 2:** Global patient engagement (PE) and patient experience data (PED) references from peer-reviewed journals included in the analysis

Publication title	Region/country	References on PE	References Integrating PE + PED	References on PED
Precision oncology medicines and the need for real world evidence acceptance in health technology assessment: Importance of patient involvement in sustainable healthcare [45]		✓	✓	✓
The immaturity of patient engagement in value-based healthcare—A systematic review [46]	Global	✓	✓	✓
A HTA of what? Reframing through including patient perspectives in health technology assessment processes [47]	Europe	✓	✓	✓
Emerging healthcare interventions: Patient-Centered Outcomes Research Institute’s programmatic initiative [48]	US	✓		✓
Patient and citizen participation at the organizational level in health technology assessment: An exploratory study in five jurisdictions [49]	International	✓		
Real-world evidence: Experiences and challenges for decision making in Latin America [50]	LATAM	✓		
An evaluation of managed access agreements in England based on stakeholder experience [51]	UK	✓		
Health technology assessment 2025 and beyond: Lifecycle approaches to promote engagement and efficiency in health technology assessment [52]	Global	✓	✓	✓
Expanding health technology assessment towards broader value: Ireland as a case study [53]	Ireland	✓	✓	✓
Using social media research in health technology assessment: Stakeholder perspectives and scoping review [54]	International			✓
Regulatory, health technology assessment and company interactions: The current landscape and future ecosystem for drug development, review and reimbursement [2]	International			✓
Novel approach to decision making for orphan drugs [55]	Czechia	✓	✓	✓
Stakeholder perspectives on cooperation in the clinical and nonclinical health technology assessment domains [56]	Europe	✓		
The value and impact of health technology assessment: Discussions and recommendations from the 2023 Health Technology Assessment International Global Policy Forum [57]	Global	✓		
Patient engagement for the development of equity-focused health technology assessment (HTA) recommendations in the digital era [58]		✓		
Patient engagement and patient experience data in regulatory review and health technology assessment: A global landscape review [10]	Global	✓	✓	✓
Use of real-world evidence for drug regulatory decisions in China: current status and future directions [59]	China			✓
Recommendations on the selection, development, and modification of performance outcome assessments: A good practices report of an ISPOR Task Force [60]	Unspecified—Global			✓
A roadmap for increasing the usefulness and impact of patient-preference studies in decision making in health: A good practices report of an ISPOR Task Force [61]	Global	✓	✓	✓
An interview with the Food and Drug Administration about draft patient-focused drug development guidance 3: Selecting, developing, or modifying fit-for-purpose clinical outcome assessments [62]	US	✓		✓
Quantitative benefit-risk assessment in medical product decision making: A good practices report of an ISPOR Task Force [63]	Global	✓	✓	✓
An updated review of Food and Drug Administration warning and untitled letters for clinical outcome assessment claims between 2013 and 2021 [64]	US			✓
Illustrating emerging good practices for quantitative benefit-risk assessment: A hypothetical case study of systemic biologic treatments for plaque psoriasis [65]	EU/US			✓
Developing, selecting, and modifying performance outcome assessments [66]	Unspecified		✓	✓
A value framework to assess patient-facing digital health technologies that aim to improve chronic disease management: A Delphi approach [67]	Multinational			✓
Developing patient-centered real-world evidence: Emerging methods recommendations from a consensus process [68]		✓	✓	✓

ISPOR, The Professional Society for Health Economics and Outcomes Research; HTA, health technology assessment; LATAM, Latin American; PE, patient engagement; PED, patient experience data; PE + PED, integrated PE and PED

**Table 3:** Global patient engagement (PE) and patient experience data (PED) references from referred resources included in the analysis

Initiative	Organization/agency	Region/country	References on PE	References integrating PE + PED	References on PED
Recommendations for patient involvement in HTA in Central and Eastern European countries [69]	HTA	Central Eastern Europe	✓	✓	✓
HTA4Patients [70]	EUPATI	Europe	✓		
About EUCAPA [71]	EURORDIS-Rare Diseases Europe	Europe	✓		
Improving interpretation of evidence relating to QoL in HTA assessments of rare disease treatments [72]	Journal “The patient—patient centered outcomes research”, authorship from EU-funded Horizon 2020 IMPACT-HTA	Europe/UK		✓	✓
Webinar: Framework for characterizing impact of patient involvement in HTA [73]	HTAi Patient & Citizen Involvement in HTA Interest Group’s	Global	✓	✓	✓
Advancing use of patient evidence in decision-making [74]	DIA Global Forum	Global	✓	✓	✓
Patient engagement action plan [75]	DIA Global Forum	Global	✓	✓	✓
Patient engagement that enables regulatory decisions: Co-creating next steps in PFDD [76]	DIA Global Forum	Global	✓	✓	✓
HTA similarities and differences across 27 countries [77]	Lymphoma Coalition	Global	✓		✓
Development and early qualitative evidence of 2 novel PROM instruments to assess daily functioning in early-stage Parkinson’s [78]	SpringerOpen	Global		✓	✓
Opportunities to improve adoption of HRQoL evidence as part of the French HTA process [79]	AstraZeneca	Global			✓
Webinar: Using PED to evaluate medical interventions [80]	IQVIA Institute	North America		✓	✓
Publication: Using PED to evaluate medical interventions [81]	IQVIA Institute	North America		✓	✓
How should medical device manufacturers use patient preference information? [82]	MDIC & Edwards	North America			✓
Expert meeting: Engaging stakeholders in trial design [83]	CTTI	US	✓		
Current practices and challenges when submitting PED for FDA regulatory decisions (industry survey) [84]	Coalition of pharma companies & Comparative Health Outcomes, Policy, and Economics (CHOICE) Institute, University of Washington School of Pharmacy	US			✓

CHOICE, Coalition of Pharma Companies & Comparative Health Outcomes, Policy, and Economics; CTTI, Clinical Trials Transformative Initiative; DIA, Drug Information Association; EUCAPA, European Capacity Building for Patients; EUPATI, European Patients Academy on Therapeutic Innovation; EURORDIS, European Organisation for Rare Diseases; FDA, Food & Drug Administration; HRQoL, health-related quality of life; HTA, health technology assessment; HTAi, Health Technology Assessment International; MDIC, Medical Device Innovation Consortium; PE, patient engagement; PED, patient experience data; PE + PED, integrated PE and PED; PFDD, patient-focused drug development; PROM, patient-reported outcome measures; QoL, quality of life

### Increasing Role of PE and PED in HTA/Regulatory Processes

Our results show an increased focus on PE (Fig. 1B), with regulators and HTA bodies strengthening their use of PE methodology and issuing guidance that sets expectations and drives PED generation. The CDA recommended including patient partners throughout all stages of the drug development research process, and clearly reporting descriptions of their role and degree of involvement in submissions to regulatory/HTA organizations [27]. Recent legislation on collaborative decision-making for orphan drugs in Czechia invites PO participation in HTA submissions and patients in the Ministry of Health’s advisory board to facilitate satisfactory patient access to such treatments [55]. In this case, involving patients, POs, and healthcare professionals fostered a more holistic multi-stakeholder dialog as well as obtaining consensus in the appraisal process.

The Consumer Engagement and Education (CEE) team of the Agency for Care Effectiveness (ACE), an HTA body in Singapore, actively involves healthcare consumers in the HTA process [85] by providing mechanisms for:

- Suggesting which pharmaceuticals and health technologies should be evaluated by ACE.
- Sharing their lived experiences with various conditions and unmet needs.
- Providing feedback on educational resources co-developed by the CEE, POs, and clinicians.

In the United States, ICER’s updated (2023) Value Assessment Framework encourages drug/medical technology manufacturers to describe PED collection methods, outline patient involvement in study design, and explain how outcomes of most interest to patients were identified for clinical trials [86]. It seeks to increase PE by facilitating:

- Online sharing of patient/caregiver testimonials.
- Group discussions on lived experience with conditions.
- Virtual/in-person attendance at public meetings and compensation for the same.
- Creation of educational resources on their review process.
- Creation of a Patient Council to provide continuous oversight on the PE program [86].

Such approaches exemplify emerging good practices of HTA bodies clarifying pathways for patient/public involvement in the assessment process, thereby encouraging their participation. Although these examples demonstrate efforts to incorporate patient insights into HTA submissions in a scientifically robust manner, such input is often still considered “complementary” or “supplementary” rather than essential to the assessment process [47].

### Patient Involvement in Managed Access Agreements

Managed access agreements (MAA) can facilitate earlier patient access to investigational treatments by collecting and reviewing evidence for a holistic evaluation of the treatment [51, 77]. Using a qualitative focus group methodology, researchers identified potential negative impacts of MAA on patients and caregivers, such as:

- Regular healthcare assessments that govern access to treatment can create intense psychological pressure about their “performance” during appraisal.
- Provider decisions to withdraw treatments can have devastating impacts on affected patients.
- The required legal paperwork can be confusing and burdensome.
- Lack of communication and transparency due to commercial sensitivities during the MAA period can limit patient groups’ ability to support their members [51].

To ensure robust evidence generation (including PED) during the agreement period, the researchers advocate healthcare decision-makers and industry sponsors participating in regular communication with patients, to plan timelines and manage expectations, as well as involving them in the design and execution of these agreements.

### Increasing Importance of Real-World Data/Evidence

Several guidance documents were released in 2023 on real-world evidence (RWE) in HTA/regulatory assessments and best practices for real-world data (RWD) collection, use, and reporting—such as those developed with support from the CDA, FDA, and Australian Government Department of Health and Aged Care [3, 12, 27]. These guidance reports describe the collection of qualitative and quantitative PED by patients and their family members/caregivers, POs, disease research foundations, researchers, and drug manufacturers as RWD for regulatory submissions [3, 12].

The 2023 ICER Value Assessment Framework encourages manufacturers to describe PED collection methods (which vary across countries) and identify the outcomes most important to patients in clinical trials and RWD [86]. As part of the Cancer Moonshot program, the FDA requested additional funding to support the collection of PED and its interpretation [43]. During discussions held at the 2022 HTAi Global Policy Forum and HTAi Annual Meeting, the HTA community was urged to advance methods and frameworks for leveraging RWE [52]. The Australian Medicines Intelligence Centre of Research Excellence also recognized the value of multi-stakeholder representation (including patients and their caregivers/families) in advisory groups overseeing the use of RWE in HTA deliberations [12]. Taken together, these guidance documents identified significant opportunities for expanding RWD availability in HTA/regulatory assessments, including patient involvement in determining questions to be addressed by RWE [12].

### Integration of PE and PED

To improve reporting consistency and comparability of studies for a given condition, and to reduce risk of selective reporting bias, the CDA recognized the importance of a standardized core outcome set (COS)—an example of an effort to integrate PE and PED. The COS comprises a standardized collection of measurable outcomes that should be reported as part of all clinical trials in a given therapeutic area. It should be developed by multiple stakeholders, engaging with patients, caregivers, or POs in the development process, to measure clinical efficacy as well as outcomes important to patients for daily functioning [27]. This recommendation by CDA supports similar calls by the HTAi: discussions held at the 2022 HTAi Global Policy Forum and their subsequent Annual Meeting recommended broad, routine, scalable, and compensated patient involvement in the HTA process [57]. Discussants also encouraged the development of COSs in partnership with patients and clinicians. Mandatory use of such COSs for efficient information-sharing among HTA bodies throughout product life cycles was recommended.

The European Network for Health Technology Assessment (EUnetHTA) also emphasized the value of involving and engaging with a wide range of stakeholders (patients, caregivers, healthcare professionals, and pharmaceutical and health technology developers) in deciding patient-centered outcomes for any given therapy [28]. Their approach aims to ensure that patients and their caregivers can provide valuable evidence on outcome measures directly related to aspects of their health most meaningful to them (e.g., ability to walk from one building to another). At the same time, clinical experts can provide guidance on indirect clinical outcome measures following the therapeutic intervention (e.g., walking capacity measured by 6-Minute Walk Test) to evaluate the suitability of proposed or selected outcomes in HTA/regulatory assessments [66].

**Fig. 2:**
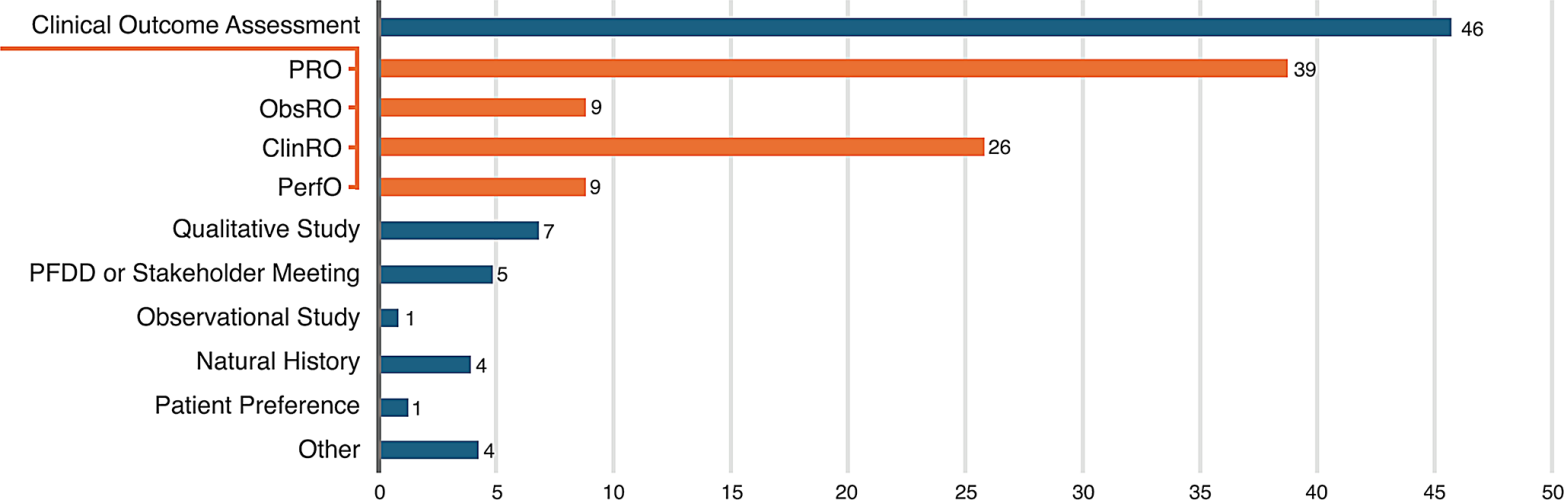
Types of PED in new drug or biologic applications approved by FDA’s Center for Drug Evaluation and Research in 2023^a^ [87]. ^a^Among 55 new drug applications and biologics license applications approved by the US FDA’s Center for Drug Evaluation and Research in 2023. Orange bars represent the types of clinical outcome assessments (COAs) included with FDA submissions. Individual submissions sometimes included more than one COA. ClinRO, clinician-reported outcomes; FDA, Food and Drug Administration; ObsRO, observer-reported outcomes; PED, patient experience data; PerfO, performance outcome; PFDD, patient-focused drug development; PRO, patient-reported outcomes

In 2023, the US FDA’s CDER approved 55 NDAs and BLAs [87], of which 46 included PED collected using a clinical outcome assessment (COA) tool (Fig. 2). Patient-reported outcome measures (PROMs) were used to collect PED in 39 of the approved submissions (71% of 2023 approvals vs. 65% in 2022). Five approvals were supported by Patient-Focused Drug Development Meeting summaries or Voice of the Patient reports. The FDA sometimes uses PED to guide benefit-risk determinations in their assessment. For example, for approval of a treatment for primary hyperoxaluria type 1 in 2023, the FDA used PED collected using PROMs as well as data from a patient stakeholder meeting, a Voice of the Patient report, and a patient community-guided cross-sectional survey on patient/caregiver preferences and disease impacts [88]. Although the FDA provided narratives on how PED contributed to benefit-risk assessments [6], quantitative PED (e.g., COA and a preference study) submitted for FDA decision-making was not always included in the benefit-risk assessment, usually because data-collection tools had not been previously validated by the FDA for use in the intended population.

### Transparency in Use of PE/PED in HTA/Regulatory Assessments

HTAs and regulatory bodies are increasingly recognizing the importance of transparency around the use of PE, PED, RWE, and the value of public trust in healthcare decision-making processes [12, 29, 53]. To foster public confidence in the safety, efficacy, and patient-centered relevance of available treatments, and to encourage continued PE, patients should be shown how their input has influenced regulatory assessments and HTAs; this demonstrates the value and impact of their contribution. To that end, in 2023 ICER launched “snapshots” or plain-language summaries in various disease/therapy areas to help educate patients on disease states, medications, the regulatory assessment process, and specific ways that PE impacted each stage of the ICER review process [39].

Despite limited practical guidance on specific processes for incorporating PE and PED in HTA submissions and regulatory processes, there has been some recent progress. For example, ICER provided recommendations on how HTA bodies could engage patients and leverage PED to improve health equity for underserved patient communities [38]. They indicated that HTA bodies should address logistical and financial barriers to direct PE for better understanding the needs of diverse patient groups, establish representation criteria for diverse patient populations in clinical trials, and refrain from calculating cost-effectiveness estimates stratified by race, ethnicity, or socioeconomic status even if clinical evidence suggests differences in the magnitude of benefit received by patient subpopulations [38]. Publishing and disseminating such guidance also keeps all stakeholders informed on how PED is used in HTA/regulatory assessments.

### Geographic Variations

**Fig. 3:**
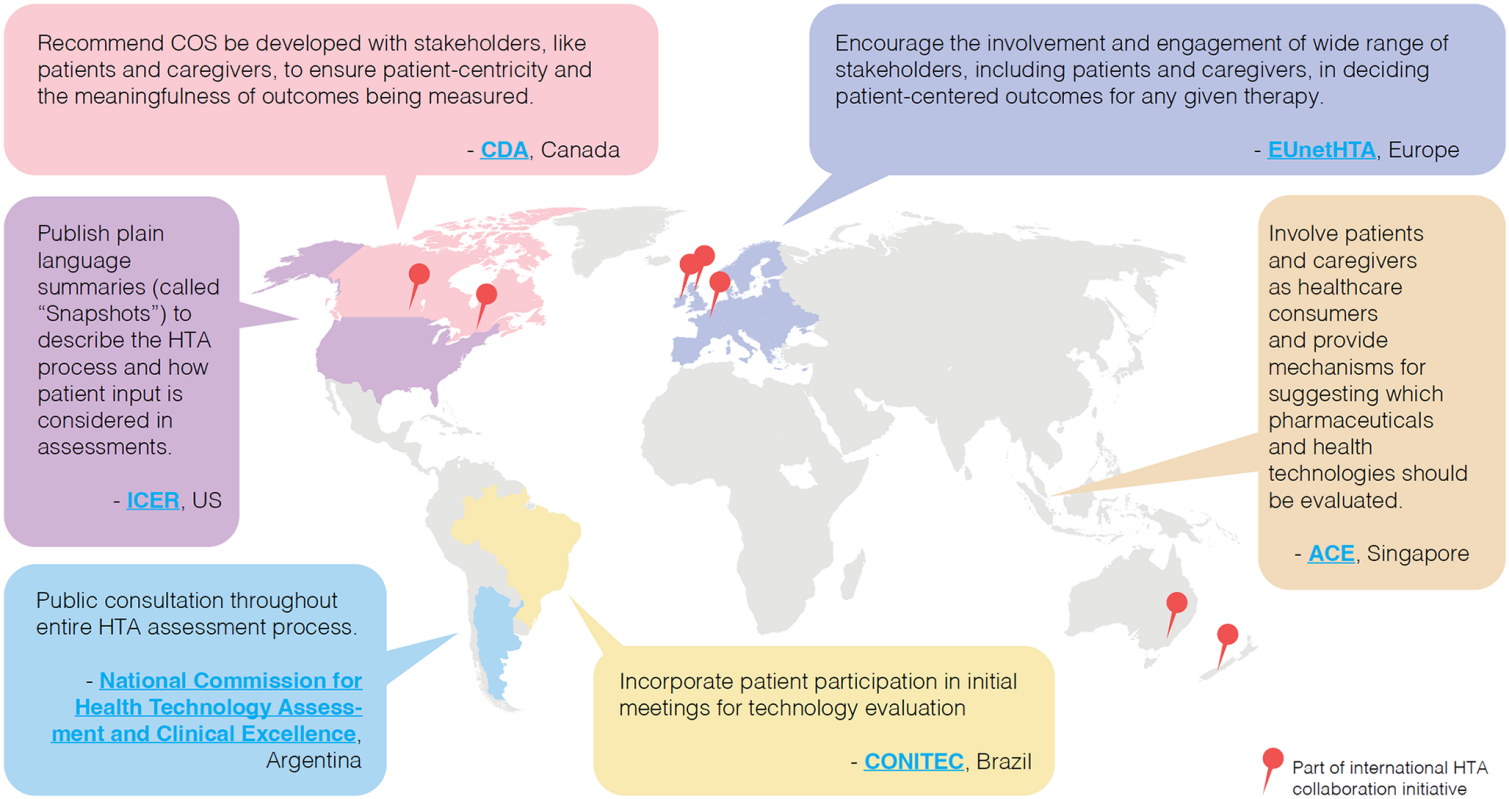
Examples of ongoing international efforts to engage patients and leverage PED in HTAs in 2023. ACE, Agency for Care Effectiveness; CDA, Canada’s Drug Agency; CONITEC, National Committee for Health Technology Incorporation; COS, core outcome set; EUnetHTA, European Network for Health Technology Assessment; HTA, health technology assessment; ICER, Institute for Clinical and Economic Review; PED, patient experience data

The Lymphoma Coalition’s 2023 HTA Workshop revealed significant time, capacity, and resource constraints faced by POs from various countries that impede their participation in the complex and resource-intensive HTA submission process [77]. Opportunities for patient involvement and mechanisms to support them also vary (Fig. 3): countries like Brazil, Germany, Japan, Singapore, Sweden, Taiwan, and Turkey encourage patient participation on health advisory boards and HTAs, whereas patients are rarely included in Korean HTA processes, reportedly due to lack of transparency and support. Countries such as Argentina consult public opinion for every step of the HTA process. Australia, Germany, Scotland, Singapore, and Wales have dedicated PE staff, committees, and councils to act as a bridge between government regulatory bodies, HTA bodies, and patients/POs [22, 77]. In contrast, countries like China generally lack guidelines or procedures to support PE in HTA/regulatory deliberations.

A high degree of collaboration exists between HTA agencies of several countries, especially in horizon scanning for new and emerging drugs/health technologies [52, 89]. For example, eight HTA bodies across Australia, Canada, and the UK collaborate to share their learnings and use shared opportunities and challenges to develop new approaches to HTA, thereby improving their individual capacities [89]. HTA agencies and government regulatory bodies in different countries could also collaborate to develop consistent guidelines and/or procedures to support PE-driven PED practices in HTA/regulatory deliberations worldwide. Such collaboration is already underway among countries in the European Union [28].

## Discussion

As regulators and HTA bodies increasingly seek to align healthcare systems with patient needs and preferences, their deliberations must be informed by the lived experiences of healthcare users. Worldwide, key decision-makers are rapidly and sustainably embracing this trend: the formal adoption of PE and PED is a relatively recent phenomenon, yet in just 12 months since our previous analysis [10], we have observed the rapidly expanding role of PE and PED in HTA/regulatory processes. This positive trend demonstrates increasing recognition of the value of PE and PED in the transformation of healthcare to create sustainable patient-centered systems fit for the future.

While both PE and PED can individually add value to HTA/regulatory assessments, it is their integrated combination that has the potential to most representatively inform such deliberations [90]. High-quality, robust PED can only be achieved by closely involving patients in its design, generation, and use. Our previous report found no published guidance from HTA/regulatory bodies on PE + PED; just a year later, we are encouraged to see an increase to nearly one-third of the HTA/regulatory references identified for 2023, but there is a continued need for more explicit direction on best practices.

Although regulators propose that patients collect and submit PED [29, 37], patients/POs can be impeded by lack of time, capacity, and resources. Pharmaceutical and medical technology companies can submit large dossiers of evidence, but POs may be constrained by word counts and strict templates that considerably limit the insights and knowledge they can share [91, 92]. Guidance for POs to generate and submit PED into regulatory/HTA processes is largely absent. There is a need to develop and improve such guidance to ensure direct PE + PED input to enhance regulatory deliberations. This guidance should be developed in partnership with the patient community. We call on researchers to systematically investigate these gaps and identify potential solutions to address them.

Close stakeholder collaboration is also needed to increase efficiencies, streamline and harmonize data collection, and decrease burden on patients [10]. PE + PED should be viewed as essential to support evidence-based decisions, rather than as optional supporting information for primary clinical outcomes data. Patients should be engaged in the design and generation of PED to ensure that domains meaningful to them are captured, as well as helping with the interpretation of PED within HTA/regulatory processes. Patient involvement at all stages can inform assessments that more accurately reflect the disease burden and treatment impacts to patients and society. Given the increasing use of RWE to support HTA/regulatory submissions, PE + PED as a key component of RWD can inform evidence generation, particularly in rare diseases, to help improve clinical and quality-of-life outcomes that are important to patients and clinicians alike. Furthermore, involving patients in an open collaborative process can help continue their impact on pharmaceutical development, possibly with continued public interest in funding healthcare programs and support for clinical trial legislation.

Development and use of a COS can help ensure the high value and patient-centricity of the outcomes being measured and reported [27], although this approach is not without challenges. As different COSs are developed for use in different contexts (e.g. clinical practice or interventional trials), interoperability of these different sets can be challenging [93]. Many existing COSs are not developed through robust methods and could be influenced by stakeholder preferences [94]. COSs may also restrict innovation in outcome measurement. Limited ability to explore new outcome measures might discourage their adoption [19]. Thus, PE-PED integration for robust evidence generation would benefit from HTA/regulatory guidance on standardization while allowing for continued innovation in development of COSs.

The impacts of PE and PED on the appraisal of evidence for pharmaceutical and medical technology development should be clearly communicated to patients and the public, as has been done through plain-language summaries. This would encourage patient participation from the inception of research questions by evidence generators, through to advising government regulatory bodies and HTA agencies on recommendations for market access, as well as build trust with the general public about the value of the end product.

We observed regional differences in the extent of PE and PED generation and use, which could ultimately impact healthcare decision-making and eventual access to interventions or reimbursement policies in different regions. Advances in patient involvement during HTA/regulatory evaluations are mostly being made in Argentina, Australia, Brazil, Europe, North America, the Philippines, Singapore, and Taiwan, albeit to varying extents [77]. Aligning with the WHO’s resolution on increased social participation in healthcare decision-making, we believe that several countries in Africa, Asia, and South America should consider adopting policies similar to some of their European and North American counterparts to generate meaningful PED and engage patients, caregivers, and POs for HTA/regulatory assessment. The regional differences support a call to action for universal alignment of sustainable best practices for PE and PED, while being sensitive to regional and cultural differences, needs, and priorities. Increasing consistency of PE and PED practices around the world will also lead to better consideration of needs and preferences of diverse patient populations. This, in turn, should encourage development of more valuable treatments and representatively inform HTA/regulatory assessments to benefit diverse groups of patients.

In this annual update to the previous landscape analysis, we expanded our methodology from including only articles referred to us by collaborators or identified via Google searches to also searching target peer-reviewed journals and HTA/regulatory body websites for relevant references published in 2023. We specifically searched for English-language guidance published in 2023 around the use of PE and PED from such authorities in various countries. Recognizing that academic research will inform the evolution of such guidance, we further included relevant peer-reviewed articles in our analysis to capture academic trends and insights into HTA/regulatory guidance. This evolution of methodology allowed us to better capture ongoing activities of HTAs and regulators as well as current thinking and analyses on the topics of PE and PED in the academic literature. Our current methodology allowed for precise tagging of source types which in turn increased transparency around current trends. For example, in the previous publication [10], the six PE + PED resources identified were all from the PFMD initiative, and we did not identify trends among HTA/regulatory bodies straight from the source. In the current manuscript, specific references discussing PE + PED published by HTA/regulatory bodies have been included.

This updated landscape review does, however, have some limitations. In the absence of a single global database, we were restricted to searching the HTA/regulatory websites of a few countries that publish in the English language and did not capture all available guidance from around the world. We limited our search to a narrow list of open access peer-reviewed journals, so other informative peer-reviewed articles on this topic might not be included in our analysis. A systematic literature review in the future may help overcome bias that may have been introduced by this methodology. Although we tried to apply a comprehensive list of search terms to these HTA/regulator and journal websites, the search-engine algorithms could have filtered out articles relevant to this review. However, we relied on the expertise of a global and diverse group of stakeholders for referrals on relevant articles published in 2023. Within these limitations, we have included all available resources at the time of writing.

We invite all stakeholders globally to join our collaborative effort on sharing learnings, best practices, resources, and insights on the universal alignment of PE-driven PED practices to inform HTA/regulatory recommendations that can benefit patients worldwide. We encourage our readers to work together to advocate for the co-creation of PED with meaningful PE, and strengthened PE approaches that offer contextualization to deliberative processes.

## Conclusion

In this update to our previously published 17-month analysis [10], we noted a higher absolute number of references from HTA/regulatory bodies worldwide that mention PE and PED, together and individually, in a 12-month period. Nearly one-third of HTA/regulatory references emphasized PE + PED, compared with none in the earlier analysis, signaling an accelerating awareness of its importance since the previous analysis period; a very welcome and encouraging trajectory.

The following key issues need to be addressed:

- Improved alignment of PE and PED practices in regulatory/HTA processes and deliberations across the world.
- Further awareness that robust PED to inform these deliberations is optimally generated with incorporation of effective PE.
- Explicit direction on PE-driven PED practices should be provided by HTA/regulatory bodies and communicated early to all stakeholders involved in evidence generation, including patients and their caregivers.
- Development of comprehensive and actionable guidance on best practices for PE and on the design, generation, analysis, and use of PED for HTA/regulatory submissions.
- Increased transparency around how HTA/regulatory bodies use such evidence in their review processes, including the impact of PED on final recommendations.

Patients should be regarded as critical stakeholders with equal standing to other collaborators and stakeholders in pharmaceutical and medical technology evidence generation as well as HTA/regulatory processes. The impact of their contributions at every step of these processes should be communicated, to ensure clear accountability and foster continued participation and holistic evidence generation. We are encouraged by the findings from this analysis, which show continued recognition of the value of the patient perspective by HTA/regulatory bodies, and anticipate more robust guidance as this important topic gains momentum in the years to come.

## Electronic Supplementary Material

Below is the link to the electronic supplementary material.

Supplementary Material 1

## Data Availability

No datasets were generated or analysed during the current study.
