# Supplementary material for: Patient Engagement and Patient Experience Data in Regulatory Review and Health Technology Assessment: Where Are We Today?
Source: Ther Innov Regul Sci. 2025 Apr 10;59(4):737–52. doi: 10.1007/s43441-025-00770-6 (PMC12181203; doi:10.1007/s43441-025-00770-6)
Supplement: Supplementary file 1 — Supplementary Material 1 [file 43441_2025_770_MOESM1_ESM.docx]

**SUPPLEMENTAL MATERIAL**

Bertelsen N, et al.^^[[1]](#footnote-1)^*^
**Patient Engagement and Patient Experience Data in Regulatory Review and Health Technology Assessment: Where Are We Today?**

**CONTENTS**

[**Fig. S1.** Numbers of resources on PE and PED published in 2023 by type^a^ 2](#_Toc188968431)

[**Table S1** Patient Focused Medicines Development team members and their roles 3](#_Toc188968432)

**Fig. S1.** Numbers of resources on PE and PED published in 2023 by type^a^


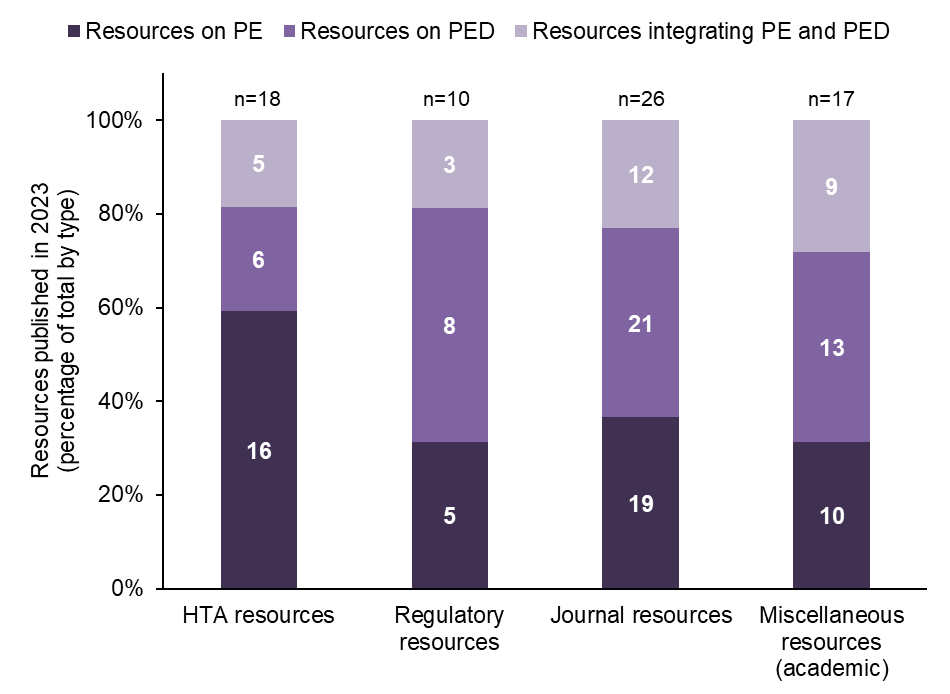
^a^Numbers within bars indicate numbers of resources on PE, PED, and integrated PE and PED for each type, numbers outside bars indicate the number of unique resources identified for each type in 2023. Numbers within bars do not add up to the numbers on top as the same resource may focus on PE, PED, and/or integrated PE and PED

HTA, health technology assessment; PE, patient engagement; PED, patient experience data

**Table S1** Patient Focused Medicines Development team members and their roles

| **Team member** | **Role** |
| --- | --- |
| Maria Marano | Analysis of referred resources |
| Nicole Wicki | Analysis of referred resources |
| Daniela Luzuriaga Ubilla | Compilation of complete list of referred resources |

1. ^*^ Corresponding author: Hayley Chapman; [hayley@thesynergist.org](mailto:hayley@thesynergist.org) [↑](#footnote-ref-1)
